# Analysis of risk factors associated with acute pancreatitis after endoscopic papillectomy

**DOI:** 10.1038/s41598-020-60941-3

**Published:** 2020-03-05

**Authors:** Eloy Taglieri, Otávio Micelli-Neto, Eduardo Aimoré Bonin, Suzan Menasce Goldman, Rafael Kemp, José Sebastião dos Santos, José Celso Ardengh

**Affiliations:** 1 Digestive Endoscopy Unit, Hospital 9 de Julho, São Paulo, SP Brazil; 2 Endoscopy Unit, Complexo Hospital de Clínicas, Universidade Federal do Paraná (UFPR); Hospital Erasto Gaertner, Curitiba, PR Brazil; 3 Department of Diagnostic Imaging, Escola Paulista de Medicina, Universidade Federal de São Paulo (UNIFESP), São Paulo, SP Brazil; 4 Department of Surgery and Anatomy, Hospital das Clínicas, Ribeirão Preto Medical School, Universidade de São Paulo (USP); Digestive Endoscopy Unit, Ribeirão Preto, SP, Brazil

**Keywords:** Bile duct cancer, Biliary tract cancer, Risk factors

## Abstract

Acute pancreatitis (AP) is a common adverse event (AE) of endoscopic papillectomy (EP). Prophylactic plastic pancreatic stent (PPS) placement appears to prevent AP. We evaluated factors associated with post-EP AP by a retrospective analysis of patients with tumors of the duodenal papilla who underwent EP from January 2008 to November 2016 at 2 tertiary care centers. Clinical, laboratory, endoscopic ultrasound parameters, and PPS placement were evaluated. Seventy-two patients underwent EP (37 men), with mean age of 60.3 (31–88) years. Mean main pancreatic duct (MPD) diameter was 0.44 (0.18–1.8) cm. Mean tumor size was 1.8 (0.5–9.6) cm. Tumors were staged as uT1N0, uT2N0, and uT1N1 in 87.5%, 11.1%, and 1.4%. Thirty-eight AEs occurred in 33 (45.8%) patients, with no mortality. Total bilirubin, tumor size, MPD diameter, and PPS placement had odds ratios (ORs) of 0.82, 0.14, 0.00, and 6.43 for AP. Multivariate analysis (PPS placement × MPD diameter) showed ORs of 4.62 (95%CI, 1.03–21.32; *p* = 0.049) and 0.000 (95%CI, 0.00–0.74; *p* = 0.042) for AP. In conclusion, patients with jaundice, large tumors, and dilated MPD seem less likely to have post-EP AP. PPS placement was associated with a higher risk of AP, which may question its use.

## Introduction

Tumors of the duodenal papilla (DP) account for 0.2 to 5% of all gastrointestinal neoplasms, and carcinoma is the most common neoplasm of the small intestine^1^. Adenoma and adenocarcinoma of the DP are the most common tumors. Adenocarcinomas have been recently accepted to originate from adenomas of the DP, and complete resection is mandatory for cure^1^. Traditionally, pancreaticoduodenectomy has been considered the preferred curative treatment option. However, the high morbidity and mortality rates associated with this procedure have prompted investigation of less invasive approaches, such as endoscopic papillectomy (EP).

Accumulating evidence indicates that EP is a safe and effective alternative to surgery for adenomas and early-stage tumors^2–6^. Although EP compares favorably with surgical treatment, the risk of EP-related adverse events (AEs) is not negligible. Even in experienced hands, AEs occur in approximately 20% of cases^3–10^. Acute pancreatitis (AP) is one of the most feared complications, occurring in approximately 10 to 15% of cases. After EP, AP is often of mild severity, requiring conservative treatment. However, 2 deaths due to necrotizing pancreatitis have been reported^5^. Studies have shown that prophylactic placement of a plastic pancreatic stent (PPS) reduces the incidence of AP after endoscopic retrograde cholangiopancreatography (ERCP) in other risk situations^5^. Prophylactic PPS placement appears to prevent obstruction of the pancreatic flow secondary to edema that is caused in the region by tumor resection using electrocautery. Studies addressing this issue are still scarce, and many of them have produced conflicting results^7,11,12^.

Endoscopic ultrasound (EUS) is the best imaging modality for investigation of the papillary region and biliopancreatic confluence. The TN staging of papillary tumors is effective and, along with the investigation of the common bile duct (CBD) and the main pancreatic duct (MPD), reinforces the indication for EP by accurately showing that the tumor is confined to the papilla^13–17^. The present study was therefore designed to evaluate factors associated with the occurrence of AP in a controlled group of patients with tumors of the DP subjected to EP.

## Materials and Methods

We conducted a retrospective controlled observational study of clinical cases staged by EUS and treated with EP with curative intent from January 2008 to November 2016 in the Digestive Endoscopy Unit of the Department of Surgery and Anatomy at the Hospital das Clínicas, affiliated with Ribeirão Preto Medical School – University of São Paulo, and at the Hospital 9 de Julho, located in São Paulo, Brazil. All patients were identified from prospectively maintained databases.

The indication criteria for EP were patients aged ≥ 18 years with a suspected diagnosis of tumor of the DP who had tumors staged as uT1 or uT2 by EUS. The tumors were diagnosed by endoscopic biopsy or by histopathological examination of the specimens after EP. Those who were not candidates for surgical treatment were included after multidisciplinary evaluation and follow-up by the surgery team. EP was contraindicated in patients with tumors staged as uT3 or uT4 or invading ≥ 1.0 cm into the CBD or MPD, and in patients undergoing endoscopic sphincterotomy for placement of plastic and/or metal stents.

The patients’ medical records were reviewed for data on age, sex, tumor type, serum bilirubin and amylase levels (before and after EP), type of tumor resection (piecemeal or en bloc), and PPS placement (yes or no).

The study was approved by the Research Ethics Committee of the Federal University of São Paulo (approval number: 1426/2017). Written informed consent was obtained from all individual participants included in the study, and the procedures were performed in accordance with the American Society for Gastrointestinal Endoscopy^7^.

### Endoscopic ultrasound staging

**Figure 1:**
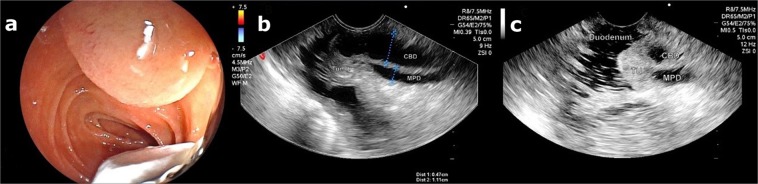
**(a)** Echoendoscope positioned in front of duodenal papilla. **(b)** EUS image showing a dilated CBD and the MPD with a “fall” next to the duodenal papilla. **(c)** The entire hypoechoic tumor is seen, with well-defined borders, not invading the CBD or MPD. Tumor staged as uT1N0.

All EUS and EP procedures were performed by experienced endoscopists, with the patient under anesthesia with propofol-controlled sedation. Patients remained hospitalized for 2 days after the procedure. Tumor staging and intraductal involvement were assessed by EUS immediately before EP. Tumors were staged according to the TNM classification. All EUS procedures were performed using a Fujifilm EG-530UT and EG-530UT2 convex array echoendoscope (FUJIFILM Medical Systems, Wayne, NJ, USA)^16^. EUS determined whether the tumor was limited to the sphincter (uT1) or invading the duodenal wall (uT2) (Table 1 and Fig. 1). EUS data on tumor stage, tumor size, and MPD and CBD diameters were recorded.

**Table 1:** Endoscopic ultrasound classification used for tumors of the duodenal papilla and respective indications according to staging.

uTN	Tumor invasion (T)/Lymph nodes (N)	Endoscopic papillectomy?
uT1	Tumor limited to the duodenal papilla	yes
uT2	Tumor invading the duodenal wall	yes
uT3	Tumor invading 2.0 cm or less into the pancreas	no
uT4	Tumor invading peripancreatic structures or adjacent organs	no
uN0	No lymph node involvement	yes
uN1	Lymph node enlargement, suspected neoplastic involvement	yes + EUS-FNA

EUS-FNA, endoscopic ultrasound-guided fine needle aspiration.

### Endoscopic papillectomy technique

**Figure 2:**
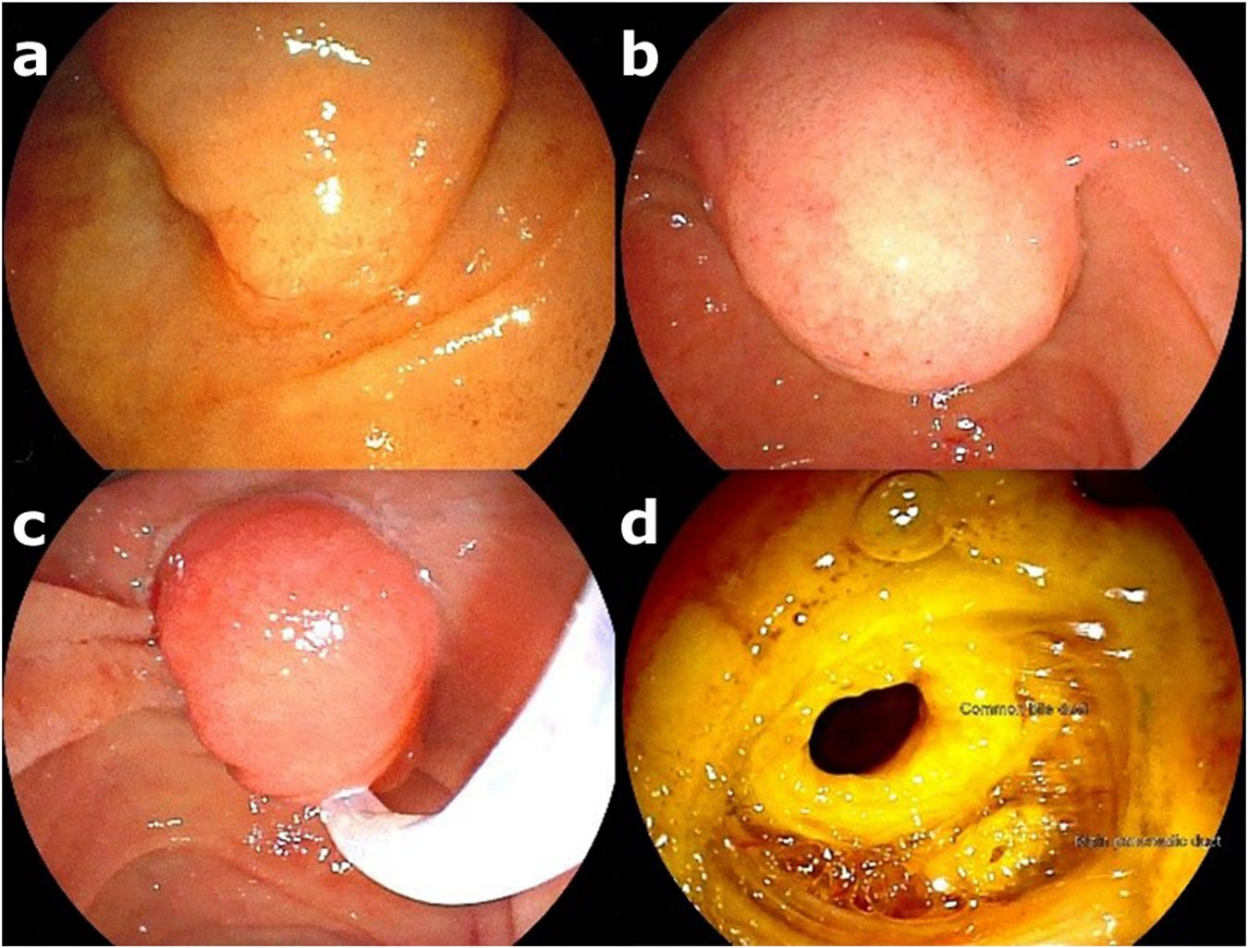
(**a)** A bulging major duodenal papilla without visible mucosal changes. **(b)** Endoscopic view of the bulging in the upper part of the papilla. **(c)** En bloc grasping of the tumor. **(d)** Post-EP resection site (note the CBD and MPD orifices).

All procedures followed the same protocol. An oval or hexagonal polypectomy snare (1.3 to 2.7 cm) (Cook Endoscopy, Winston-Salem, NC, USA) was used to grasp the entire tumor/papilla complex to be resected en bloc (preferably), being cut with monopolar current (35 W output power) without coagulation at any point during resection (WEM SS-200E electrocautery device, COVIDIEN; and Valleylab Force FX electrosurgical generator, Medtronic) (Fig. 2). Piecemeal resection was indicated only for lesions spreading laterally to the duodenal mucosa or lesions ≥ 3 cm.

A Fujinon ED-530XT8 duodenoscope (FUJIFILM Medical Systems, Wayne, NJ, USA), or even the convex array echoendoscope, was used when its position in relation to the papilla was favorable. In case of bleeding, endoscopic hemostasis was performed with epinephrine and glucose injections and/or hemoclips. Argon plasma coagulation (APC), at 60 W and flow rate of 2.0 L/min, was considered if any potential residual tissue was observed.

In cases of prophylactic stent placement, a 7- or 5-Fr Cotton-Leung PPS was placed (Cook Endoscopy, Winston-Salem, NC, USA). In each case, the PPS was individually adjusted to the MPD length at the head of the pancreas and placed after EP with the aid of a guidewire introduced into the MPD after injection of a small amount of contrast.

### Adverse events and follow-up

Patients were observed for the occurrence of AEs during the procedure, in the recovery room and for 2 postoperative days while hospitalized. AEs that occurred after discharge were recorded as reported by the patients and health professionals in subsequent follow-up visits. All AEs were documented and classified according to the period of occurrence as follows: immediate, AEs occurring within 24 hours of EP; early, AEs occurring from 1 to 30 days after EP; and late, AEs occurring after 30 days of EP^18^.

Cases of treatment failure were referred for surgical treatment. Those deemed unsuitable for surgery were treated with palliative stenting. Serum tests (amylase, lipase, among others) were performed and analyzed within 24 hours of EP.

The diagnosis of AP was defined based on the recommendations of the 2012 revised Atlanta classification^19^ requiring the presence of at least 2 of following 3 parameters: (1) clinical complaint (abdominal pain, nausea and/or vomiting); (2) serum amylase level at least 3 times greater than the upper limit of reference; and (3) evidence of characteristic findings of AP on computed tomography. Patients with increased serum amylase levels, in the absence of any other parameter for AP, were classified as having hyperamylasemia.

Follow-up included evaluation at the time of the first control endoscopy scheduled 4 weeks after EP for stent removal, endoscopic biopsies, and adjuvant therapy if necessary. All patients were seen and examined every 3 months during the first year, and then annually until completing 5 years of follow-up.

### Anatomic pathology

Resected specimens were sent for histopathological examination. Patients with a diagnosis of invasive malignancy (infiltration of the duodenal submucosa) or intramucosal malignancy with nontumor-free margins were referred for surgery if clinically indicated.

### Therapeutic and clinical success

Treatment was considered successful if complete resection was achieved without invasive adenocarcinoma and no recurrence was observed within 1 year of follow-up. Clinical success was defined as improvement of symptoms.

### Statistical analysis

To explore risk factors associated with post-EP AP, especially PPS placement, 2 logistic regression models were used. In the first model, univariate logistic regression analyses were performed to test the association of placement/non-placement of PPS with the occurrence of AP, and to explore possible associations between the development of AP and clinical features, such as total, direct, and indirect bilirubin and amylase levels before EP, tumor size and MPD diameter measured by EUS. In the second model, multivariate logistic regression analysis was adjusted for placement/non-placement of PPS by age, sex, tumor size, and MPD diameter, the latter two measured by EUS.

Changes in Akaike’s information criterion (AIC) and McFadden Pseudo R^2^ values were calculated for assessing the multivariate model fitting. The significance of the coefficients of the model was obtained by the Wald statistic. All analyses were performed in R (version 3.3.2 for Mac), and the level of significance was set at *p* ≤  0.05.

### Patient consent and ethical approval

The study was approved by the Research Ethics Committee of the Federal University of São Paulo (approval number: 1426/2017). Written informed consent was obtained from all individual participants included in the study, and the procedures were performed in accordance with the American Society for Gastrointestinal Endoscopy.

## Results

### Patient characteristics

Seventy-two patients with tumors of the DP who underwent EP between January 2008 and November 2016 were included in the study. Mean patient age was 60.3 (31–88) years, with a male-to-female ratio of 1:1 (37 men/35 women). Papillary tumors were an incidental finding during digestive endoscopy with bulging of the papillary region or some mucosal irregularities in 22 cases (30.5%). Jaundice was the most common symptom, accounting for 35 (48.6%) cases, followed by abdominal pain in 6 (8.3%) cases and AP in 4 (5.5%) cases. Mean total bilirubin was 5.57 (0.3–40) mg/dL. The characteristics of the study population are shown in Table 2.

**Table 2:** Characteristics of patients undergoing endoscopic papillectomy (n = 72).

Characteristics	Total
Mean age, years (min, max)	60.3 (31–88)
Sex, n (%)	
Male	37 (51.4)
Female	35 (48.6)
Symptoms, n (%)	
Jaundice	35 (48.6)
Asymptomatic	22 (30.5)
Abdominal pain	6 (8.3)
Acute pancreatitis	4 (5.5)
Cholangitis	3 (4.2)
Anemia	2 (2.8)
Mean bilirubin and amylase (pre-EP)	
TB, mg/dL (min, max)	5.57 (0.3–40)
DB, md/dL (min, max)	3.83 (0.07–26)
IB, mg/dL (min, max)	1.82 (0.6–14)
Amylase, U/L (min, max)	131.61 (9–1460)
Endoscopic ultrasound	
Mean MPD diameter, cm (min, max)	0.44 (0.18–1.80)
Mean CBD diameter, cm (min, max)	0.9 (0.3–2.1)
uTN classification, n (%)	
uT1N0	63 (87.5)
uT1N1	1 (1.4)
uT2N0	8 (11.1)
Mean tumor size, cm (min, max)	1.8 (0.5–9.6)
Tumor resection, n (%)	
Piecemeal	13 (18.1)
En bloc	59 (81.9)
PPS placement, n (%)	
Yes	20 (27.8)
No	52 (72.2)
Tumor histology, n (%)	
Adenocarcinoma	30 (41.6)
Adenoma	25 (34.7)
Neuroendocrine tumor	6 (8.3)
Papillary hyperplasia	4 (5.5)
Lipoma	2 (2.8)
Normal papilla	2 (2.8)
Stromal tumor of the duodenal papilla	1 (1.4)
Lymphoma	1 (1.4)
Hamartoma	1 (1.4)

TB, total bilirubin; DB, direct bilirubin; IB, indirect bilirubin; MPD, main pancreatic duct; CBD, common bile duct; PPS, plastic pancreatic stent.

### Endoscopic ultrasound

The mean MPD diameter was 0.44 (0.18–1.80) cm. The mean CBD diameter was 0.9 (0.3–2.1) cm. Of 72 tumors, 63 (87.5%) were staged as uT1N0, 8 (11.1%) as uT2N0, and 1 (1.4%) as uT1N1. Mean tumor size was 1.8 (0.5–9.6) cm (Table 2).

### Endoscopic papillectomy and final diagnosis

En bloc resection was possible in 59 (82%) patients. A prophylactic PPS was placed in 20 (28%) patients. The final diagnosis was adenocarcinoma in 30 (41.6%) cases, followed by adenoma in 25 (34.7%) and neuroendocrine tumor in 6 (8.3%) (Table 2).

### Adverse events

A total of 38 AEs occurred in 33 (45.8%) patients. Four patients had more than 1 AE. AP was the most common AE, accounting for 17 (23.6%) cases, followed by bleeding in 15 (20.8%) cases, perforation in 2 (2.8%), stenosis in 2 (2.8%), cholangitis in 1 (1.4%), and acute cholecystitis in 1 (1.4%). The 2 post-EP stenosis cases resulted from AP about 4 months after resection. There were no deaths in this series.

Twenty (27.8%) patients developed AEs during or immediately after EP: bleeding in 11 (15.3%); AP in 7 (9.7%); and perforation in 2 (2.8%). Between 1 and 30 days after EP, 14 (19.4%) AEs occurred: AP occurred in 10 (13.8%) patients and bleeding in 4 (5.5%). Late AEs occurred in 4 (5.5%) patients: stenosis of the MPD in 2 (2.8%); cholangitis in 1 (1.4%); and acute cholecystitis in 1 (1.4%) (Table 3).

**Table 3:** Occurrence of adverse events after endoscopic papillectomy (n = 72 patients).

Adverse events*	n	Immediate (≤ 24 h)	Early (1–30 days)	Late (> 30 days)
Acute pancreatitis	17	7	10	—
Bleeding	15	11	4	—
Perforation	2	2	—	—
Stenosis	2	—	—	2
Cholangitis	1	—	—	1
Cholecystitis	1	—	—	1
Total	38^†^	20 (27.8%)	14 (19.4%)	4 (5.5%)

Immediate: adverse events occurring within 24 hours of the procedure; Early: those occurring in the first 30 days; Late: those occurring after 30 days.

*p = 0.186.

^†^38 adverse events occurred in 33 (45.8%) patients.

### Risk factors for the occurrence of acute pancreatitis after endoscopic papillectomy

Univariate analysis revealed that bilirubin, tumor size, MPD diameter, and PPS placement were associated with the occurrence of AP. Patients with increased levels of total, direct, and indirect bilirubin were less likely to have AP, with odds ratios (ORs) of 0.82 (95% confidence interval [CI], 0.66–0.95; *p* = 0.044), 0.78 (95%CI, 0.58–0.95; *p* = 0.045), and 0.43 (95%CI, 0.17–0.83; *p* = 0.036), respectively. No statistically significant association was found between the occurrence of AP and serum amylase levels (OR = 1.00; 95%CI, 0.99–1.00; *p* = 0.152), nor between AP and sex (OR = 0.58; 95%CI, 0.19–1.76). However, tumor size and MPD diameter had ORs of 0.14 (95%CI, 0.03–0.55) and 0.000 (95%CI, 0.00–0.16), respectively. These results show that both parameters are inversely proportional to the risk of developing AP, but it was not possible to measure the exact tumor size and MPD diameter for the occurrence of AP. Patients who received a PPS had an OR of 6.43 (95%CI, 1.97–21.01) in relation to those who did not receive a prophylactic PPS for AP (Table 4).

**Table 4:** Risk factors for the occurrence of acute pancreatitis after endoscopic papillectomy with or without prophylactic plastic pancreatic stent (PPS) placement.

	OR (95%CI)	Adjusted OR (95%CI)	*P (Wald’s test)*
Bilirubin (mg/dL)			—
TB	0.82 (0.66–0.95)	—	—
DB	0.78 (0.58–0.95)	—	—
IB	0.43 (0.17–0.83)	—	—
Amylase (U/L)	1.00 (0.99–1.00)	—	—
**Sex (n/%)**			
Female (35/48.6%)	—	—	—
Male (37/51.4%)	0.58 (0.19–1.76)	0.32 (0.07–1.38)	0.126
Age	0.96 (0.92–1.04)	0.98 (0.93–1.04)	0.502
Tumor size (cm)	0.14 (0.03–0.55)	0.41 (0.08–2.04)	0.059
MPD diameter (cm)	3.072e-04 (2.567e-07–0.078)	0.00 (0.00–0.74)	**0.042**
PPS placement (AP n/%)	—	—	—
No (7/9.6%)	—	—	—
Yes (10/50%)	6.43 (1.97–21.01)	4.62 (1.03–21.32)	**0.049**

MPD, main pancreatic duct; OR, odds ratio; CI, confidence interval; TB, total bilirubin; DB, direct bilirubin; IB, indirect bilirubin; AP, acute pancreatitis;

McFadden Pseudo R^2^ = 0.311.

Multivariate analysis showed that only MPD diameter and PPS placement were directly associated with the occurrence of AP. MPD diameter was inversely proportional to the likelihood of developing AP when a PPS was placed, with an OR of 0.000 (95%CI, 0.00–0.74; *p* = 0.042). When adjusted for MPD diameter and other potential confounding factors, those receiving a PPS were more likely to develop AP, with an OR of 4.62 (95%CI, 1.03–21.32; *p* = 0.049) (Table 4).

In the multivariate model, we performed an analysis with potential confounding variables that could influence the occurrence of AP, such as sex, age, and tumor size. However, when associated with PPS placement and MPD diameter, none of these factors produced significant changes in the risk of AP (Table 4). A comparison of the characteristics of patients undergoing PPS placement in relation to the occurrence of AP was then performed, and the results are shown in Table 5.

**Table 5:** Comparison of the general characteristics of patients undergoing PPS placement in relation to the occurrence of AP.

	No AP after EP	AP after EP	*p*
	Mean (SD)	Mean (SD)	
Patients with PPS	10 (100.00)	10 (100.00)	
Age, years	60.60 (14.54)	52.20 (13.12)	0.192
Sex, % male	7 (70.0)	5 (50.0)	0.648
CBD diameter, cm	0.84 (0.35)	0.73 (0.39)	0.523
MPD diameter, cm	0.40 (0.10)	0.31 (0.07)	0.035
Tumor size, cm	2.09 (2.69)	1.15 (0.51)	0.289
Pancreatitis	0.00 (0.00)	1.00 (0.00)	<0.001
Bleeding	1.80 (0.42)	1.80 (0.42)	1.000

PPS, plastic pancreatic stent; AP, acute pancreatitis; EP, endoscopic papillectomy; SD, standard deviation; CBD, common bile duct; MPD, main pancreatic duct.

## Discussion

After the first series of EPs performed by Binmoeller *et al*.^[.2^, many others have also demonstrated the effectiveness of EP^3,4,20–22^. Endoscopic biopsy has shown low sensitivity and accuracy, ranging from 40 to 89%^23,24^. This limited accuracy provides the first positive support for indication of EP, that is, obtaining adequate material for histological examination without jeopardizing the possibility of a subsequent surgical approach^4^.

EP is performed not only with curative intent but also for diagnosis, staging (T), and guidance on appropriate treatment. The EP techniques published in the literature are heterogeneous and, given the rarity of papillary tumors, it may be difficult to achieve standardization^4,7^. Performing ERCP before EP is unnecessary, as it is a predisposing factor for the occurrence of AEs; in addition, papillotomy is a contraindication for en bloc resection of the DP. Performing pancreatic sphincterotomy after EP^7^ is also unnecessary^25^. Biliary sphincterotomy^7^ is indicated only in patients with choledocholithiasis. In this case, caution should be exercised in its use because of the increased risk of perforation, which may occur because of altered anatomical parameters^25^, as observed in one of our patients.

In the present series, EUS was performed immediately before EP to assess peripapillary involvement, duodenal wall invasion, CBD, and MPD. The use of EUS helps to more accurately indicate EP, which may be wider and deeper. EUS allowed us to systematize EP so that the approach was uniform. This helped us with information for the positioning of the polypectomy snare to enable en bloc resection of the papilla. EUS also allowed evaluation of lymph nodes, which were observed in one of our patients with a tumor staged as uT1N1 whose final diagnosis was somatostatinoma with the presence of 2 lymph nodes detected by EUS, in whom EUS-guided fine needle aspiration was performed in the same EP session^9^.

The success of EP was determined by complete tumor resection with free margins on histological examination. In the present series, a success rate of 64% was obtained, with en bloc resection being performed in 82% of these EPs. Previous studies have reported success rates ranging from 29 to 92%^20,24,26,27^. These results are related to factors such as variations in techniques, tumor size, and experience level of endoscopists.

EP is associated with an increased risk of AEs, ranging from 8 to 35%, with bleeding and AP being the most common^9^. In our series, the AE rate was 45.8%, with AP occurring in 23% of cases and bleeding in 21%. All episodes of bleeding were controlled endoscopically. Performing sphincterotomy adds a risk of perforation, with an incidence of 0 to 8%^22^. Careful examination of the resection defect after EP can identify a perforation, which can be treated endoscopically. Endoscopic treatment may vary according to the site of occurrence and includes biliary stenting in cases of perforation of the cranial border or clipping in the caudal border. There were 2 cases of perforation in our series (2.8%). One occurred after sphincterotomy performed after EP for removal of gallstones, which was treated endoscopically by biliary stenting, and the other occurred after disinsertion of the papilla during EP in a patient who had undergone Billroth II gastrectomy; the patient was referred for surgery immediately after EP^28^.

The most concerning post-EP AE is AP, with an incidence of 5 to 15%^3–5,9^, most often presenting with mild severity and good outcome^29^. Placement of a PPS has shown positive results in patients at high risk of developing AP after ERCP^5^, and some authors have adopted this approach after EP^4^. However, most data come from retrospective studies, thus hindering proper systematization of the resection technique and comparison of results. There is only one prospective, randomized, controlled trial in which EP was performed with and without PPS placement^8^. However, the institutional review board interrupted the trial after the occurrence of 3 cases of AP in the unstented group^8,30^. This interruption can be regarded as premature, because it compromised the study power for statistical analysis.

Although the usefulness of prophylactic PPS placement has been demonstrated in some studies^4,8,30^, the precise role of PPS placement in preventing AP after EP remains to be determined, and its use has been questioned in recent series that failed to demonstrate a reduction in AP risk^7,11,12^. Chang *et al*.^11^ published a retrospective study of 82 patients who underwent EP, with PPS placement in 54 and no stent placement in 28 (patients with patulous MPD opening after EP), reporting an incidence of AP of 10.5% in the stented group vs 7.14% in the unstented group, but with no significant difference between the groups (*p* = 1.00).

In the present study, stented patients had a higher risk of developing AP than unstented patients (OR = 6.43; 95%CI, 1.97–21.01). Theoretically, these results suggest that patients undergoing PPS placement are 6.43 times more likely to develop AP. This finding differs from the literature^3,5,6,8,21,30–33^. It should be noted that this is a retrospective, nonrandomized study, which precludes causal inferences about the relationship between the procedures. However, this study provides some evidence for questioning routine PPS placement after EP. The incidence of AP was 50% in our patients who received a PPS, against 13% in those who did not. The trauma caused by post-EP PPS placement in a normal-caliber MPD may have been the cause of AP. Placing a guidewire in the MPD and performing EP over the guidewire^31–33^ facilitate PPS insertion, and this approach has been proposed by some authors. Even so, it is not an easy procedure to perform^34^. Repeated attempts to cannulate the MPD, followed by PPS placement, may cause local trauma, which appears to be the etiology of AP.

This is the first study to assess tumor staging, CBD and MPD diameter by EUS immediately before EP and evaluate whether this parameter has any influence on the occurrence of AP. We attempted to define a minimum MPD diameter from which PPS placement would provide some benefit by reducing the occurrence of AP. In fact, we did observe that the greater the MPD diameter, the lower the likelihood (OR < 1) of developing AP (OR = 0.000; 95%CI, 0.00–0.16), supporting the hypothesis that post-EP AP is caused by MPD obstruction. However, we were unable to quantify the exact MPD diameter to prevent AP after PPS placement. For this purpose, a study with a larger number of patients and designed with this type of evaluation is required.

We observed that increased indirect bilirubin levels (OR = 0.43; *p* = 0.036) and tumor size (OR = 0.14; *p* = 0.005) were associated with reduced risk of AP. Jaundice as a secondary sign of chronic papillary obstruction and tumor size reflect a greater obstruction to the biliopancreatic flow, leading to chronic dilatation of the MPD. This reinforces the theory that post-EP AP is related to pancreatic duct obstruction.

One aspect to be considered is the relationship between the risk factors for post-EP AP. In the univariate analysis, there was a relationship between MPD diameter, PPS placement, and tumor size; however, the association between tumor size and AP did not remain significant in the multivariate analysis. One of the main arguments in favor of PPS placement is the results obtained in ERCP studies^5^. However, EP and ERCP differ markedly in their approaches, since at least part of the sphincter of Oddi is resected in EP, so their correlation should not be attempted. This is evident in our analysis, where, in addition to the association of PPS placement with a higher risk of AP, known risk factors for post-ERCP AP, such as age and sex^35^, were not associated with AP in our series.

In conclusion, data from the present study show that EP is a safe procedure, with low mortality but high morbidity. Performing EP immediately after EUS is important for resection of the DP tumor. EUS, besides being a valuable staging tool, can accurately determine tumor site and size as well as MPD diameter, a risk factor for the occurrence of AP. Patients with jaundice, large tumors, and dilated MPD appear to be less likely to have an episode of AP after EP, and prophylactic PPS placement seems to be dispensable in most cases because of its association with a higher risk of developing AP. Further studies are certainly warranted to support these conclusions.
